# Investigating the Role of Skin Autofluorescence in Gestational Diabetes Mellitus: A Systematic Review

**DOI:** 10.3390/ijms26073022

**Published:** 2025-03-26

**Authors:** Bianca-Margareta Salmen, Delia Reurean-Pintilei, Dan Trofin, Cristiana-Elena Durdu, Alexandra-Cristina Neagu, Roxana-Elena Bohiltea

**Affiliations:** 1 Doctoral School, ‘Carol Davila’ University of Medicine and Pharmacy, 020021 Bucharest, Romania; bianca-margareta.mihai@drd.umfcd.ro (B.-M.S.); cristiana-elena.durdu@rez.umfcd.ro (C.-E.D.); 2 Department of Medical-Surgical and Complementary Sciences, Faculty of Medicine and Biological Sciences, “Stefan cel Mare” University, 720229 Suceava, Romania; 3 Department of Diabetes, Nutrition and Metabolic Diseases, Consultmed Medical Centre, 700544 Iasi, Romania; trofin.dan@umfiasi.ro; 4 Department of Biomedical Sciences, Faculty of Medical Bioengineering, University of Medicine and Pharmacy “Grigore T. Popa” Iasi, 700454 Iasi, Romania; 5 Department of Obstetrics and Gynaecology, Filantropia Clinical Hospital, 011132 Bucharest, Romania; dr.bohiltea@gmail.com; 6 Department of Audiology, ‘Maria Sklodowska Curie’ Children’s Emergency Clinical Hospital, 077120 Bucharest, Romania; aaaneagu30@yahoo.com; 7 Department of Obstetrics and Gynaecology, ‘Carol Davila’ University of Medicine and Pharmacy, 020021 Bucharest, Romania

**Keywords:** gestational diabetes mellitus, skin autofluorescence, pregnancy, advanced glycation end products, macrosomia

## Abstract

Gestational diabetes mellitus (GDM) is a pregnancy-specific condition that can cause serious complications for both the mother and the fetus. Preventing these complications requires optimum glycemic control. Skin autofluorescence (SAF) is a non-invasive and innovative method that evaluates the levels of advanced glycation end products, markers of hyperglycemia, that could aid in the optimum management of GDM-complicated pregnancies. This systematic review aims to assess SAF’s potential utility in the prediction of short-term and long-term outcomes in GDM. Following the Preferred Reporting Items for Systematic Reviews and Meta-Analyses (PRISMA) methodology, with the protocol identifier CRD42024559012, we used “(skin autofluorescence OR SAF) AND (gestational diabetes mellitus OR GDM)” as a search criterion on the PubMed, Scopus, and Web of Science databases. After a rigorous selection process, we included five articles, which evaluated SAF values and GDM, SAF and pregnancies complicated by diabetes mellitus, and SAF and macrosomia. GDM diagnosis varies due to the different approaches among the major guidelines, leading to variations in interpretation and diagnostic thresholds. Across studies, this variability contributes to inconsistent SAF values. As a standardized and objective marker, SAF could provide a uniform criterion, improving GDM management. Further research is needed to validate its clinical utility.

## 1. Introduction

According to the American Diabetes Association, gestational diabetes mellitus (GDM) is a specific form of diabetes mellitus (DM) that is diagnosed in the second half of pregnancy, typically in the second or third trimester. The diagnosis is made after excluding other types of DM such as pre-existing overt DM, type 1 DM (T1DM), or other forms of DM that could complicate pregnancy [1]. GDM presents a prevalence varying between 2% and 38%, depending on the different demographic characteristics of the specific population [2]. This rising prevalence has been linked to several factors, such as an increased maternal age at conception, an increased maternal body mass index, and the increased global incidence of obesity [3].

While well-known risk factors for GDM include a family history of DM, previous GDM, increased maternal age, prior macrosomic births, a prepregnancy body mass index ≥ 30 kg/m^2^, and insulin resistance (IR)-associated conditions such as polycystic ovary syndrome [4,5,6,7]), genetic predisposition may also play a role. Several genetic polymorphisms have been associated with increased GDM risk, particularly in the TCF7L2 and MTNR1B genes. Variants as rs12243326, rs4506565, rs12255372, rs7901695, and rs7903146 in the TCF7L2 gene have showed a higher susceptibility to GDM and have been linked to impaired insulin secretion [8,9]. The MTNR1B rs10830963 G allele affecting the melatonin receptor function has been observed in connection with an increased need for insulin therapy in obese pregnant women despite adequate nutritional intervention [10]. At the molecular level, GDM-related insulin deficiency interferes with serotonin uptake in the trophoblast and leads to elevated serotonin level in the placenta, vasoconstriction, and impaired placental perfusion [11,12].

GDM poses a variety of immediate and long-term consequences for both the mother and the fetus. Poor glycemic control during pregnancy often leads to short-term outcomes such as macrosomia and large-for-gestational-age fetuses and newborns (defined as having a weight above the 90th percentile for the specific gestational age) [13], polyhydramnios [14], gestational hypertension, preeclampsia [15], and neonatal morbidities such as hypoglycemia, hypocalcemia, hyperbilirubinemia, hypomagnesemia, cardiomyopathy, and respiratory distress [16,17], and it is also associated with fetal mortality, especially stillbirth [18]. Regarding long-term effects, infants born from mothers with GDM present higher risks of developing obesity [19] or type 2 DM (T2DM) [20]. Patients with GDM have a 10-fold increased risk of developing T2DM in the future compared to healthy pregnant women [21]. They are also more likely to develop metabolic syndrome three months postpartum [22,23] and cardiovascular (CV) disease (CVD) independently of its association with T2DM [24]. In addition, a large cohort study [25] concluded that over a follow-up period of 30 years, patients with GDM have a higher crude mortality rate (1.74 versus 1.49, hazard ratio of 1.25, 95% confidence interval (CI) (95% CI) 1.11–1.41) than patients without GDM in their obstetrical history, primarily due to the presence of CVD.

Currently, GDM monitoring is primarily conducted through the self-monitoring of blood glucose (SMBG) and continuous glucose monitoring systems (CGMSs) [26,27]. SMBG provides only intermittent glucose data and may become burdensome for patients as it requires multiple invasive blood glucose measurements daily. In contrast, CGMS offers the complete image of glucose fluctuations and real-time insights into trends and variability patterns. Several studies, such as the CONCEPTT trial, have demonstrated the benefits of CGMS in pregnancies complicated by DM reporting a reduction in neonatal hypoglycemia, macrosomia, and the need for neonatal intensive care unit admissions [28]. Despite all these advantages, CGMS remains underutilized in routine GDM management, partly due to cost and accessibility limitations.

Given these challenges, there is a need for alternative and accessible yet standardized biomarkers for metabolic dysfunction. Advanced glycation end products (AGEs), which accumulate during hyperglycemia, serve as stable indicators of glucose exposure over time. Quantifying AGEs through their fluorescent properties is performed by a validated and non-invasive technique called skin autofluorescence (SAF), which has been shown to provide a potential alternative or complement existing monitoring methods [29,30,31]. Studies suggest that SAF correlates with glucose metabolism disturbances and may predict both short- and long-term complications, including macrosomia [32,33].

Considering SAF’s attributes, i.e., its non-invasive nature, ease of use, and reproducibility, our review aims to comprehensively evaluate its potential utility in predicting both short- and long-term outcomes in GDM while the current literature on this topic is highly heterogeneous. Our objective is to synthesize existing evidence to improve the clinical management of pregnancies complicated by GDM, thereby reducing maternal–fetal morbidity and mortality. Additionally, we aim to enhance postnatal care for mothers, addressing the heightened metabolic and CV risks associated with GDM.

## 2. Results

To facilitate a clearer synthesis of the included studies, we summarized the main findings in Table 1, where we have included information on the author, country of origin, study period, sample size, cohort grouping, gestational weeks (GW) of SAF measurement, type of DM, SAF model, baseline SAF levels, and adverse pregnancy outcomes for each study.

The observational study conducted by De Ranitz-Greven et al. [34] in 2012 examined the relationship between SAF levels in pregnancies complicated by GDM or pre-existing DM and adverse pregnancy outcomes. The study found that SAF values decreased over time in normal pregnancies whereas in pregnancies complicated by DM, SAF levels were higher (Table 1). However, elevated SAF levels did not appear to influence adverse pregnancy outcomes.

The same group conducted another analysis [35], investigating the potential of using the AGE Reader and SAF as screening tools for GDM. The study assessed whether SAF levels were elevated in patients with GDM compared to pregnant individuals without DM. The authors concluded that SAF levels did not significantly differ at the time of GDM diagnosis compared to controls, likely due to the short duration of hyperglycemia during pregnancy. An additional noteworthy finding was the variation in SAF values across ethnicities; for instance, lower SAF levels were observed in white European pregnant patients with GDM.

Maury et al. [36] evaluated 252 patients, including two-hundred-and-thirty pregnant women with DM—two hundred with GDM and thirty with pregestational DM (PDM) (twenty-one with T1DM and nine with T2DM)—compared to twenty-two nonpregnant, nondiabetic controls. The authors reported an 11% increase in SAF values in women with PDM compared to those with GDM. They concluded that SAF levels showed a statistically significant increase (*p* for trend = 0.008) in patients with a history of hyperglycemia, defined as having had previous GDM, the birth of a macrosomic infant (weighing > 4000 g), or GDM diagnosed early in pregnancy (before the 24th GW). Additionally, the association was stronger in pregnant women meeting two or three hyperglycemia criteria, even after adjusting for age (*p* = 0.02). The authors summarized that SAF may serve as a marker of metabolic memory, reflecting long-term hyperglycemia exposure in pregnant women.

The 2019 study by Foussard et al. [33] analyzed the relationship between macrosomia and SAF values in pregnant women. The findings revealed that mothers of macrosomic infants exhibited an over-11% increase in SAF values. Using multivariate logistic regression, a 1-arbitrary-unit (AU) increase in SAF was associated with an odds ratio (OR) of 4.13 for macrosomia (95% CI: 1.46–11.71), indicating statistical significance. Among patients with GDM, a 3.8-fold increased likelihood of delivering a macrosomic newborn was observed (95% CI: 1.20–12.00). A multivariate model further demonstrated a strong association between SAF values and macrosomia after adjusting for insulin status (OR: 4.32; 95% CI: 1.52–12.30). Additionally, higher HbA1c levels were recorded in mothers who delivered macrosomic infants compared to those who did not (5.58 ± 0.91% vs. 5.30 ± 0.47%; *p* = 0.004), underlining the link between maternal SAF and macrosomia (OR: 3.27; 95% CI: 1.03–10.42). Parity also emerged as an important factor, with women of parity ≥ 1 showing a significant association between SAF values and macrosomia (OR: 4.88; 95% CI: 1.10–21.67). Furthermore, in women with parity ≥1 and a history of macrosomia, the relationship between SAF and macrosomia remained consistent (OR: 3.38; 95% CI: 1.16–9.82).

Cosson et al. published an observational case–control study [32] examining AGE levels in women with different glycemic statuses during pregnancy. After adjusting for age and ethnicity, the proportion of women with AGE levels above the mean +1 standard deviation (SD) was significantly higher across groups: no GDM (41.7%), regular GDM (45.8%), early GDM (54.8%), and DM in pregnancy (100%) (*p* = 0.032, before and after ethnicity adjustment). Similarly, the proportions of women with AGE levels exceeding the mean +2 SD were notable: 8.3%, 10.4%, 25.8%, and 50%, respectively (*p* = 0.021 before adjustment and *p* = 0.045 after ethnicity adjustment). The authors confirmed their hypothesis that elevated fasting plasma glucose levels detected early in pregnancy may reflect undiagnosed dysglycemia prior to conception. They further suggested that SAF levels indicate that early GDM could result from an intermediate glycemic state between normoglycemia and prediabetes existing before pregnancy.

## 3. Discussion

Despite GDM being a major health concern associated with serious maternal and fetal consequences, and the availability of non-invasive predictive tools like SAF, there have been few studies investigating SAF’s role in predicting, diagnosing, and preventing adverse pregnancy outcomes.

### 3.1. AGEs and Their Relevance to Pregnancy

The process of glycation is a spontaneous, enzyme-independent reaction in which reducing sugars, such as glucose, fructose, or their derivates, are interacting with free amino groups from proteins, DNA, and lipids. This is a natural phenomenon that develops gradually under normal physiological conditions, yet is augmented in hyperglycemia, oxidative stress (OS), and IR states. These are elements known to contribute to the characteristic profile for GDM and other pregnancy-related complications, such as preeclampsia and intrauterine growth restriction. In GDM, increased glucose levels contribute to the accelerated production and accumulation of AGEs. Additionally, AGEs can originate from exogenous sources such as via dietary intake from foods that are fried, highly processed, or prepared at high temperatures, being rich in AGEs [37]. As AGE levels rise, they induce cellular stress and trigger injury, ultimately resulting in pathological endothelial dysfunction [38].

AGEs have a capacity for crosslinking with macromolecules and reshaping both their structure and function. By binding to their specific receptor, i.e., the receptor for AGEs (RAGE), they generate a sequence of pro-oxidative and pro-inflammatory signals. In this context, a progression of atherosclerosis, an increase in vascular resistance, and elevated CV morbidity have been demonstrated in patients with higher AGE levels [39,40]. Additionally, SAF has shown significant potential in DM screening and several studies have stated its value in predicting CV risk [41,42]. However, there is a lack of evidence regarding its associations with several specific CV outcomes, and the impact of on these associations remains uncertain [43]. Consequently, SAF as a non-invasive marker of AGE accumulation has shown significant potential for DM screening, with numerous studies highlighting its value in predicting CVD risk [41,42]. However, evidence regarding its association with specific CV outcomes remains inconsistent, and the influence of various factors on these associations is still not fully understood [43].

In the specific context of GDM, this condition is also a state that has been associated with an increased risk for overall CVD [44]. With respect to these agents’ impact on fertility, the accumulation of AGE has been long linked with the negative impact on folliculogenesis in insulin-resistant OS stages [45,46]. RAGE is expressed also on the placental and vascular endothelium. The endothelial disfunction stemming from this interaction may negatively influence utero-placental blood flow. AGEs may disrupt placental angiogenesis, altering the expression of the vascular endothelial growth factor and placental growth factor [47]. The placental surface area for nutrient and gas exchange is therefore impaired due to these modifications [48,49]. A high first-trimester serum AGE level was found to be associated with adverse perinatal outcomes [50]. Elevated AGE levels in GDM signal increased OS and chronic low-grade inflammation. This exacerbates IR and may shadow long-term metabolic programming in the offspring, casting a long-lasting impact [51,52,53,54].

### 3.2. Heterogenous Diagnosis of GDM

The latest American Diabetes Association (ADA) guidelines emphasize that screening for dysglycemia is crucial and recommend glucose testing for women planning pregnancy, particularly in the presence of risk factors such as being overweight; having insufficient exposure to physical activity; being diagnosed with atherogenic dyslipidemia, high blood pressure, or a previous diagnosis of GDM or prediabetes; belonging to predisposed ethnic groups; or presenting IR [55]. It is recommended to assess all individuals of childbearing potential for undiagnosed prediabetes or DM, either before conception or during the first prenatal visit. Early pregnancy screening, before 15 GW, is aimed at detecting abnormal glucose metabolism as higher values may increase the risk of adverse pregnancy and neonatal outcomes, increase the need for insulin therapy, or lead to a later diagnosis of GDM. Early dysglicemia is defined by specific diagnostic criteria such as fasting plasma glucose levels of 110–125 mg/dL or A1C levels between 5.9% and 6.4%. Between 24 and 28 GW in the absence of previously diagnosed DM or early abnormal glucose metabolism, routine screening for GDM is recommended. After delivery, individuals with GDM should be offered prediabetes or DM screening at 4–12 weeks postpartum using a 75-g oral glucose tolerance test (OGTT). Lifelong monitoring is advised following the with periodic screening every 1–3 years to assess the risk of developing prediabetes or DM. These measures are aimed to improve maternal and fetal outcomes and mitigate long-term metabolic risks [54]. Currently, two strategies are used for diagnosing GDM: the one-step and the two-step approach. Both methods have been studied for their impact on maternal and offspring outcomes; however, a consensus on a uniform diagnostic method has yet to be established, and long-term outcome studies are ongoing [55,56,57,58,59,60,61]. When analyzing the articles included in this review, we found the same heterogeneity in the specific procedures for GDM diagnosis as outlined in current guidelines, reflecting the lack of a uniformly accepted diagnostic method and resulting in differences in strategies and criteria employed across studies. In the study published by de Ranitz-Greven et al. [35], GDM was diagnosed using a 100 g OGTT conducted between 20–32 GW. Another study by the same group [36] employed a two-step diagnostic approach: first, the 50 g glucose challenge test was performed in pregnant patients, followed by a 100 g OGTT conducted between 24 and 28 GW, using the ADA-recommended cut-offs in those with abnormal values [55]. Maury et al. [36] utilized the 75 g OGTT between 24 and 28 GW to diagnose GDM. In contrast, the studies by Foussard et al. [33] and Cosson et al. [32] differentiated between early and late (or regular) GDM. Early GDM was defined by a fasting plasma glucose value of ≥0.92 g/L and <1.26 g/L [33] or ≥5.1 mmol/L [32] during the first trimester. Late GDM was diagnosed using a 75 g OGTT performed between the 24th and 28th GW [33] while regular GDM was diagnosed using the same 75 g OGTT but conducted after 22 GW [32]. This non-uniformity in GDM diagnostic approaches may mask important differences or similarities in specific expected outcomes and may complicate the ability to draw strong and relevant conclusions about the role of SAF in GDM diagnosis and prediction. Additionally, it may impact prognostics, including adverse pregnancy events, SAF levels, and metabolic markers. While this inconsistency in testing protocols mirrors real-world clinical practice, it limits the applicability of the findings to guide in standardized guidelines. Consistency and comparability across research studies and clinical settings would improve the quality of evidence and care in GDM.

### 3.3. Interpopulation Variability

Among the key demographic variables influencing SAF, age and ethnicity have been consistently identified. Two studies have confirmed a statistically significant correlation between SAF values and increasing age [35,36]. In a study by de Ranitz-Greven et al. [35], SAF values increased by 0.02 AU per year (*p* < 0.001), regardless of the presence of GDM. Ethnicity also plays a role in SAF variability. De Ranitz-Greven et al. [35] identified significantly lower SAF values among white European patients with GDM (*p* < 0.001), suggesting that ethnic background may influence SAF readings. This variability may partially explain inconsistencies in the literature regarding AGEs and GDM. For instance, a 2016 study by Bartakova et al. [62], which examined the Czech population (predominantly Caucasian), reported significantly higher serum carboxymethyl-lysine (a dominant AGE) levels in GDM patients compared to healthy controls (*p* = 0.000403). In contrast, Lobo et al. [63] investigated the Euro-Brazilian population and found no statistically significant difference in serum fluorescent AGE concentrations between GDM and control groups (*p* > 0.05). These findings align with data on nonpregnant individuals, indicating that South Asian populations exhibit a slightly more pronounced increase in SAF values with age compared to other ethnic groups [63]. Given that SAF values naturally rise with age and vary across different ethnic backgrounds, it is crucial to establish specific reference ranges tailored to diverse populations [64].

SAF has the potential to be a valuable diagnostic and prognostic tool in various health-related conditions [65], provided it becomes part of routine clinical practice [41,66,67]. However, several challenges exist in its clinical interpretation. One key issue is the lack of standardized, population-specific cut-off values—while some recommendations exist, further development and validation are required [68]. SAF levels naturally increase in a linear trend with age but are significantly exacerbated in conditions including hyperglycemia, obesity, CVD, chronic kidney disease, cancer, and cognitive impairment [65,69,70,71,72]. Additionally, high-fat and highly processed foods contribute to increased AGE accumulation, further influencing SAF levels [73,74]. Other factors, including smoking and darker skin phototypes, are also associated with higher SAF values, emphasizing the need for population-specific considerations in SAF interpretation [64,75].

Nearly two decades ago, Lutgers et al. [76] established cut-off values for SAF in healthy adults and individuals with T2DM. In 2014, Klenovics et al. [77] reported distinct SAF values for the Slovakian population compared to Dutch individuals, highlighting the need for demographically specific reference data. More recently, Martínez-García et al. proposed age-group-specific SAF reference values for healthy Spanish adults [70]. While several studies have reported cut-off values for individuals with CVD [78,79,80] and DM [42] in specific populations, such data remain scarce for GDM. This highlights an important gap in the current literature rather than a neglected area as research on SAF is expanding rapidly. While HbA1c remains a widely used marker for glucose regulation, recent studies have questioned its predictive value in GDM outcomes for both the mother and child. Since GDM typically manifests in the third trimester, HbA1c does not adequately capture this relatively short hyperglycemic window. In this context, SAF presents an opportunity to offer additional insights beyond traditional glucose markers. Unlike HbA1c, which reflects average glucose levels over previous months, SAF provides a non-invasive, real-time assessment of cumulative OS and glycation processes, factors that may play a more direct role in GDM-related complications. While the implementation of SAF in routine clinical settings requires further evaluation in terms of cost-effectiveness and training, its potential for immediate risk assessment makes it a promising area of study.

Expanding this research will help refine SAF’s role in clinical practice and ensure its reliability as a risk assessment tool in GDM and other metabolic conditions.

### 3.4. GDM-Complicated Pregnancy Outcome and SAF

In this review, we noticed a notable scarcity of information in the literature regarding the possible predictive value of the specific method using SAF and the outcome of pregnancies complicated by GDM. The need for more evidence into investigating SAF as a tool for assessing both maternal and offspring health statuses remains evident. With regard to the association between SAF values and GDM pregnancies, opposite results were found.

The first study by de Ranitz-Greven et al. [34] showed no significant correlation between SAF values and adverse pregnancy outcomes, including complications, rates of large-for-gestational-age neonates, or cesarean delivery. This lack of association was attributed by the authors to the relatively mild and short-term hyperglycemia observed in GDM, which may not have been sufficient to significantly increase SAF levels or contribute to adverse prognoses. In contrast, the study by Foussard et al. [33] delivered important evidence in relation to the prognostic value of SAF in GDM. The protocol used the 75 g OGTT between 24 and 28 GW and demonstrated that each 1 AU increase in SAF was associated with a 4.13-fold higher risk of macrosomia (95% CI: 1.46–11.71) in their overall population. More specific, in the GDM subgroup, they identified a significant correlation, with an OR of 3.8 (95% CI: 1.20–12.00, *p* = 0.02). Furthermore, they underlined that the association between elevated SAF levels and macrosomia persisted after adjusting for maternal HbA1c levels, parity, and other hyperglycemia-related variables. These data suggest that SAF is a measure of the cumulative exposure to hyperglycemia and may serve as a marker for adverse fetal outcomes, macrosomia in particular.

The methodological differences between these studies, including GDM diagnostic criteria, timings of SAF measurement, and sample sizes, may explain the disparity in the presented results. For instance, using alternative diagnostic values for GDM may potentially identify different populations with varying degrees of hyperglycemia. Furthermore, Foussard et al.’s multivariate analysis, accounting for additional risk factors, may have strengthened the observed associations, whereas the de Ranitz-Greven study had limited power that could have reduced its ability to detect smaller but possibly clinically relevant correlations [33,34,35].

### 3.5. OS and Brain Implications

Excess weight and IR are frequently associated with GDM. It has been observed that IR impairs mitochondrial respiration, and early signs of cellular aging and mitochondrial dysfunction [81,82,83] are shown in mesenchymal stromal cells derived from human umbilical cords of patients with GDM. Furthermore, patients with preeclampsia exhibit increased AGE levels [83,84], thus reinforcing the role of OS in pregnancy-related complications. A hyperglycemic environment has been linked to the formation of reactive oxygen species (ROS) by upregulating specific biochemical pathways, leading to increased OS. Additionally, women are becoming pregnant at older ages and often have associated conditions such as hypertension, exposure to unhealthy environments, pro-inflammatory diets, and excess weight—whether in the form of obesity or as unfavorable adipose tissue distribution [83,85,86,87]. Each of these factors individually contributes to OS, but together, they may have a compounding effect, promoting increased ROS and free radical accumulation. One of the most concerning consequences of maternal OS is its impact on fetal brain development. Maternal adiposity is associated with increased leptin production, hyperinsulinemia, and hyperglycemia, leading to fetal hyperglycemia and excessive insulin secretion. This, in turn, triggers inflammation, IR, and the downregulation of insulin receptors and glucose transporters in the fetal brain. These disruptions compromise critical neurodevelopmental pathways in the hippocampus and cortex, regions essential for learning and memory formation. Furthermore, dysfunctional leptin signaling and OS-induced inflammation lead to microglial dysfunction, impairing neuroimmune regulation and metabolic plasticity, which may have long-term cognitive and behavioral consequences in offspring [83,88].

In this context, assessing SAF as a biomarker for AGEs underscores its potential in monitoring OS and its systemic implications for GDM complications, including neurodevelopmental risks. OS plays an important role in sustaining metabolic and vascular dysfunction by accelerating AGE accumulation, which, in turn, amplifies neuroinflammation and endothelial dysfunction. Therefore, SAF—being a non-invasive measure of AGE deposition—is considered an indicator of cumulative oxidative damage over time. Additionally, the activation of the RAGE further exacerbates inflammation and endothelial dysfunction, potentially providing valuable insights into maternal and fetal health risks. Consequently, the relationship between SAF and OS in GDM pathophysiology warrants further investigation—not only regarding peripheral complications (e.g., polyneuropathy and mononeuropathy, such as carpal tunnel syndrome) but also in understanding risks to fetal development and brain function [89,90,91].

Increasing evidence suggests that OS is a key factor in cognitive deficits associated with GDM as it may lead to neurodevelopmental impairments in children. Since pregnancy is a critical window for brain development, oxidative damage during this stage may have long-lasting effects on neuronal function, potentially increasing the risk of cognitive decline and neurodegenerative processes later in life [87]. Elevated levels of ROS can disrupt neuronal homeostasis, triggering inflammatory pathways that facilitate neurodegenerative mechanisms. The relationship between SAF, OS, and brain function in GDM is therefore underscored by the impact of AGEs on neuronal health as AGEs contribute to OS and inflammation, potentially leading to neuronal dysfunction and cognitive impairments [92,93,94]. Beyond its impact on neurodevelopment, GDM-related OS can also contribute to hematological complications. For instance, GDM can lead to fetal polycythemia, which may promote a procoagulant state and increase the risk of neonatal stroke [95]. The association between SAF and fetal health is further emphasized by the link between maternal AGE accumulation and adverse fetal outcomes. Studies indicate that higher SAF levels, beyond their association with macrosomia, are correlated with an increased risk of delivery complications and serve as an additional risk factor for other encephalopathy-related conditions [96].

### 3.6. Future Perspectives

Monitoring SAF could be a valuable instrument for providing insights into the metabolic status of pregnant women at risk for different dyslycemia ranges, or with overt GDM, potentially guiding interventions for improving maternal and fetal outcomes. It emerges as a promising non-invasive biomarker for assessing metabolic health in pregnant women with GDM. There are other markers such as HbA1c for glycemic control. Unfortunately, HbA1c remains non-reliable due to the increased red blood cell turnover, a physiologic phenomenon, having a descending trend throughout pregnancy [97], SAF impresses with the advantage of being a technique with a short learning curve, non-invasiveness, and reproducibility, as well as the benefit of possibly establishing homogenous GDM diagnosis and prognosis criteria worldwide. The single limitation is the cost of the machine. SAF’s correlation with glycemic control and potential to predict complications highlights the need for further research into its clinical applications. Future studies should focus on exploring its utility in routine clinical practice for managing GDM and its complications, and, if the results prove positive correlations between SAF values and the risk of developing DM complications, establishing standardized SAF measurement protocols should be the next step.

### 3.7. Strengths and Limitations

One of the primary limitations of our review has been the limited number of the included studies, which has reflected the relatively sparse research available on the topic of SAF in GDM. This scarcity emphasizes the limited but potentially emerging body of research on this topic, highlighting both its potential clinical relevance as well as the need for further high-quality investigations. Through the selection process, we identified only five eligible studies published between 2012 and 2019, yet this reflects the rigorous inclusion criteria rather than a definitive decline in scientific interest. However, the heterogeneity in diagnostic criteria, and in the study designs and findings, creates the context for standardized methodologies and larger, multicentric studies to clarify SAF’s role in GDM screening and pregnancy outcomes. Another key limitation relates to sample sizes as some of the included studies had included relatively small cohorts, with this aspect potentially affecting the statistical power of their findings. Additionally, the direct comparability of results is complicated taking into consideration the variability in gestational age at the time of SAF measurement as SAF levels may fluctuate across pregnancy stages. As previously stated, there has been a recognized lack of standardized, population-specific reference values, which further adds to the challenge of SAF interpretation in clinical practice. Moreover, SAF measurements are influenced by skin reflectance, particularly in individuals with darker skin tones, which could affect its applicability across diverse populations [34].

Despite these limitations, this systematic review represents, to our knowledge, the first dedicated exploration of SAF in GDM, emphasizing the need for further research rather than signaling a loss of scientific momentum. The five studies included in this review included reasonable patient numbers, yet their heterogeneity underscores the necessity of future research with uniform diagnostic criteria and prospective validation. SAF, as a non-invasive, user-friendly tool, holds promise for integration into routine clinical practice for GDM risk stratification. However, future studies should focus on larger, well-structured trials to provide a more conclusive assessment of its clinical value. By addressing these research gaps, SAF could potentially become a valuable tool for refining GDM diagnosis and predicting adverse pregnancy outcomes, advancing our understanding of metabolic stress during pregnancy. To contribute to addressing these research gaps, our collective is currently conducting an ongoing study exploring the prognostic value of SAF and CGM in maternal and child outcomes in GDM.

## 4. Materials and Methods

For our study, we conducted a reproductible protocol following the recommendations of Preferred Reporting Items for Systematic Reviews and Meta-Analyses (PRISMA) for the systematic review protocol checklist (protocol number: CRD42024559012). In addition, the Population, Intervention, Comparison, Outcome, and Study Design (PICOS) strategy was used in order to guide our research principle to obtain a comprehensive, beneficial, and systematic literature analysis. Firstly, we used the following criterion for our search: “(skin autofluorescence OR SAF) AND (gestational diabetes mellitus OR GDM)”. We identified 116 articles (52 on the Web of Science, 9 on the Scopus database, and 55 on the PubMed database). Secondly, we assessed that the studies resulted by the search engine were according to our inclusion criteria, i.e., only full-text original articles, randomized control trials, and clinical trials, published in English, in the last ten years (from 1 January 2014 to 17 November 2024), conducted only on the adult human population. The exclusion criteria covered other types of articles than original ones, such as letters to editors; reviews and comments along with meta-analyses; studies published more than 10 years previously; those involving other populations than adult human populations; and those in other languages than English.

**Figure 1:**
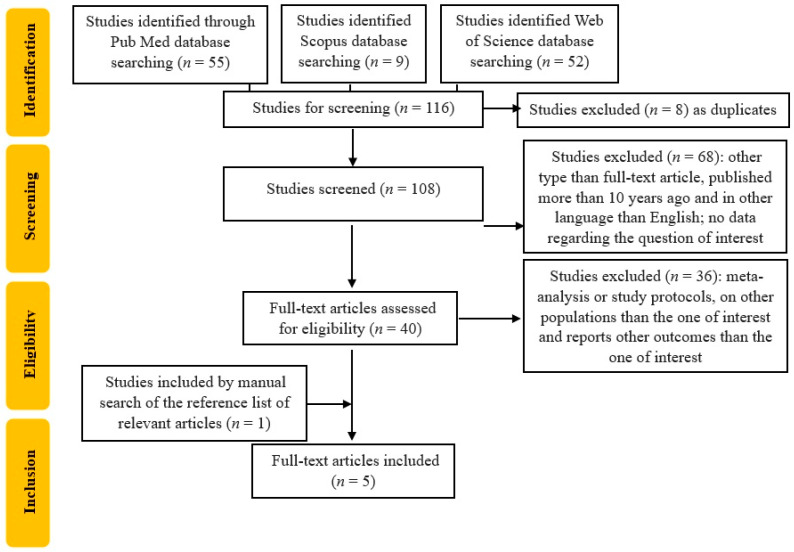
Flowchart of the study selection process.

We further searched for duplicates and excluded 8 articles, resulting in a final of 40 included studies. Two researchers (B-MS and C-ED) individually accomplished the screening process with regard to finding relevant articles. If any disagreements occurred, they were discussed and managed by a third reviewer (DRP). The selection process is represented in Figure 1. We included studies with SAF evaluation in pregnant women with GDM, so the articles that assessed serum AGEs in pregnant women with GDM, which did not include data about SAF, were excluded, resulting in a total of 4 included articles. Due to the limited number of publications regarding our subject of interest, we also completed a manual search of the references in order to establish whether there were other potentially valuable articles missed after using our search strategy, so we added one more article, resulting in a total of 5 articles.

## 5. Conclusions

This systematic review examined the potential of SAF as a non-invasive measure for AGEs and AGEs’ associations with maternal and fetal health indicators in GDM. However, varying diagnostic criteria, populations, and methodologies have contributed to inconsistent findings across studies. As long as the diagnosis of GDM lacks homogeneity between countries due to the variety of recommendations of the different professional societies guidelines, there will remain unclear criteria. In this direction, SAF could be a solution to homogenize GDM diagnosis and materno-fetal prognosis. In certain cohorts, SAF demonstrated predictive value for adverse outcomes like macrosomia, with elevated SAF values significantly increasing the associated risk. Conversely, other studies reported no significant relationship between SAF and pregnancy complications, creating the need for research standardization. Larger, multicenter trials are essential to validate SAF as a reliable predictive tool in GDM, refining risk stratification and maternal–fetal care. At present, evidence remains insufficient to support its routine clinical application, highlighting the necessity for further investigation and integration into established GDM management protocols.

## Figures and Tables

**Table 1:** Summary of the included studies.

First Author, Publication Year	Country	Study Period	Sample Size: Groups	GW Measurement	DM Type	SAF Model	Baseline SAF Level (AU)	Adverse Pregnancy Outcome Parameter	Outcome	*p* Value
de Ranitz-Greven et al. [34], 2012	The Netherlands	April 2010–December 2011	155: 79 GDM 21 PDM (13 T1DM, 8 T2DM) 55 without DM	weeks 26–29, 30–33, 34–37, and >38 GW; during the first postpartum visit (8 weeks postpartum).	GDM, T1DM, T2DM	AGE Reader (DiagnOptics Technologies BV, Groningen, The Netherlands)	PDM 1.93 Controls 1.75	Any Complication	OR 1.6 (0.56–4.7)	NS
								LGA	OR 1.3 (0.31–1.7)	
								CS	OR 1.5 (0.55–4.3)	
de Ranitz-Greven et al. [35], 2012	The Netherlands	April–December 2010	124: 60 GDM 44 without DM 20 with an abnormal value at OGTT	During 100 g-OGTT (24–28 GWs) or within the first 3 weeks after OGTT	GDM	AGE Reader (DiagnOptics Technologies BV, Groningen, The Netherlands	GDM 1.74 Controls 1.76	Increased maternal age	With every year, SAF increases with 0.02 AU	*p* < 0.001
								Ethnicity (lower SAF values in white Europeans with GDM)	1.6 AU vs. 1.9 AU	*p* < 0.001
Maury et al. [36], 2015	France	November 2011–November 2012	252: 200 GDM 30 PDM (21 T1DM, 9 T2DM) 22 control group (nonpregnant and without DM)	24–30 GWs	GDM, T1DM, T2DM	AGE ReaderTM; DiagnOptics BV, Groningen, the Netherlands	GDM 1.77 PDM 1.97 Control group 1.6	SAF higher in patients with 2 or 3 criteria for hyperglycemia *	β = 0.154; β = 0.011 after adjusting for age	*p* = 0.02; *p* = 0.009
								Increasing age	β = 0.013	*p* = 0.0019
								Fasting plasma glucose	β = 0.073	*p* = 0.03
								1-h OGTT value	β = 0.032	*p* = 0.02
								SAF was higher in GDM patients with history of hyperglycemia	0.10 AU higher	*p* = 0.04
								SAF was higher in pregnant women vs. controls	1.80 ± 0.35 AU vs. 1.6 ± 0.32 AU	*p* = 0.009
								SAF was higher in PDM than GDM	0.20 AU higher	*p* = 0.003
Foussard et al. [33], 2019	France	2011–2015	343: 39 PDM 95 early GDM 209 late GDM	PDM: 21.9 ± 8.2 Early GDM: 22.7 ± 7.2 Late GDM: 29.0 ± 3.4	GDM, PDM	AGEs-reader (DiagnOptics BV, Groningen, the Netherlands)	Not specified	Macrosomia (whole population) vs. no macrosomia	2.03 ± 0.30 vs. 1.80 ± 0.34	*p* < 0.0001
								Macrosomia (GDM)	OR: 3.80; 95% CI: 1.20–12.00	*p* = 0.02
Cosson et al. [32], 2018	France	August 2015–July 2016	188: 62 early GDM 48 GDM 8 DIP 70 controls	Not specified	GDM, DIP	AGE Reader (DiagnOptics BV, Groningen, The Netherlands	GDM: 1.99 ± 0.47 Early GDM: 2.11 ± 0.48 DIP: 2.42 ± 0.34	No GDM	1.79 ± 0.32 AU	NS
								GDM	1.99 ± 0.47 AU	NS
								Early GDM	2.11 ± 0.48 AU	NS
								DIP	2.42 ± 0.34 AU	*p* = 0.015; *p* = 0.021 after adjusting for age; *p* = 0.021 after adjusting for age and ethnicity

GW—gestational week; DM—diabetes mellitus; PDM—pre-existent diabetes mellitus; NS—not significant; OGTT—oral glucose tolerance test; AU—arbitrary units; LGA—Large for gestational age; CS—cesarean section; OR—odds ratio, followed by confidence interval; * hyperglycemia—personal history of previous GDM, a newborn with macrosomia (more than 4 kg), or GDM discovered before 24 GW; early GDM—first trimester serum fasting glucose between 0.92 and 1.26 g/dL; late GDM—diagnosed following 24–28 GWs 75 g-OGTT; macrosomia—newborn with weight > 4000 g; DIP—diabetes in pregnancy.

## Data Availability

Data sharing is not applicable to this article.

## Abbreviations

The following abbreviations are used in this manuscript:

ADA	American Diabetes Association
AGEs	Advanced Glycation End Products
AU	Arbitrary Unit
CGMS	Continuous Glucose Monitoring systems
CI	Confidence Interval
CV	Cardiovascular
CVD	Cardiovascular Disease
DM	Diabetes Mellitus
GDM	Gestational Diabetes Mellitus
GW	Gestational Weeks
IR	Insulin Resistance
OGTT	Oral Glucose Tolerance Test
OR	Odds Ratio
OS	Oxidative Stress
PDM	Pregestational Diabetes Mellitus
RAGE	Receptor for AGEs
ROS	Reactive Oxygen Species
SAF	Skin Autofluorescence
T1DM	Type 1 Diabetes Mellitus
T2DM	Type 2 Diabetes Mellitus

## References

[1] American Diabetes Association Professional Practice Committee (2024). 2. Diagnosis and Classification of Diabetes: Standards of Care in Diabetes-2024. Diabetes Care.

[2] Bilous R.W., Jacklin P.B., Maresh M.J., Sacks D.A. (2021). Resolving the Gestational Diabetes Diagnosis Conundrum: The Need for a Randomized Controlled Trial of Treatment. Diabetes Care.

[3] Feig D.S., Hwee J., Shah B.R., Booth G.L., Bierman A.S., Lipscombe L.L. (2014). Trends in incidence of diabetes in pregnancy and serious perinatal outcomes: A large, population-based study in Ontario, Canada, 1996–2010. Diabetes Care.

[4] Committee on Practice Bulletins—Obstetrics (2018). ACOG Practice Bulletin No. 190: Gestational Diabetes Mellitus. Obstet. Gynecol..

[5] Hedderson M.M., Gunderson E.P., Ferrara A. (2010). Gestational weight gain and risk of gestational diabetes mellitus. Obstet. Gynecol..

[6] Kim C., Liu T., Valdez R., Beckles G.L. (2009). Does frank diabetes in first-degree relatives of a pregnant woman affect the likelihood of her developing gestational diabetes mellitus or nongestational diabetes?. Am. J. Obstet. Gynecol..

[7] Hedderson M.M., Williams M.A., Holt V.L., Weiss N.S., Ferrara A. (2008). Body mass index and weight gain prior to pregnancy and risk of gestational diabetes mellitus. Am. J. Obstet. Gynecol..

[8] Huerta-Chagoya A., Vázquez-Cárdenas P., Moreno-Macías H., Tapia-Maruri L., Rodríguez-Guillén R., López-Vite E., García-Escalante G., Escobedo-Aguirre F., Parra-Covarrubias A., Cordero-Brieño R. (2015). Genetic determinants for gestational diabetes mellitus and related metabolic traits in Mexican women. PLoS ONE.

[9] Papadopoulou A., Lynch K.F., Shaat N., Håkansson R., Ivarsson S.A., Berntorp K., Agardh C.D., Lernmark Å., DiPiS Study Group (2011). Gestational diabetes mellitus is associated with TCF7L2 gene polymorphisms independent of HLA-DQB1*0602 genotypes and islet cell autoantibodies. Diabet. Med..

[10] Firneisz G., Rosta K., Al-Aissa Z., Hadarits O., Harreiter J., Nádasdi Á., Bancher-Todesca D., Németh L., Igaz P., Rigó J. (2018). The *MTNR1B* rs10830963 Variant in Interaction with Pre-Pregnancy BMI is a Pharmacogenetic Marker for the Initiation of Antenatal Insulin Therapy in Gestational Diabetes Mellitus. Int. J. Mol. Sci..

[11] Li Y., Hadden C., Singh P., Mercado C.P., Murphy P., Dajani N.K., Lowery C.L., Roberts D.J., Maroteaux L., Kilic F. (2014). GDM-associated insulin deficiency hinders the dissociation of SERT from ERp44 and down-regulates placental 5-HT uptake. Proc. Natl. Acad. Sci. USA.

[12] Red-Horse K., Zhou Y., Genbacev O., Prakobphol A., Foulk R., McMaster M., Fisher S.J. (2004). Trophoblast differentiation during embryo implantation and formation of the maternal-fetal interface. J. Clin. Investig..

[13] Venkatesh K.K., Lynch C.D., Powe C.E., Costantine M.M., Thung S.F., Gabbe S.G., Grobman W.A., Landon M.B. (2022). Risk of Adverse Pregnancy Outcomes Among Pregnant Individuals with Gestational Diabetes by Race and Ethnicity in the United States, 2014–2020. JAMA.

[14] Pilliod R.A., Page J.M., Burwick R.M., Kaimal A.J., Cheng Y.W., Caughey A.B. (2015). The risk of fetal death in nonanomalous pregnancies affected by polyhydramnios. Am. J. Obstet. Gynecol..

[15] Usami T., Yokoyama M., Ueno M., Iwama N., Sagawa N., Kawano R., Waguri M., Sameshima H., Hiramatsu Y., Sugiyama T. (2020). Comparison of pregnancy outcomes between women with early-onset and late-onset gestational diabetes in a retrospective multi-institutional study in Japan. J. Diabetes Investig..

[16] Page J.M., Allshouse A.A., Cassimatis I., Smid M.C., Arslan E., Thorsten V., Parker C., Varner M.W., Dudley D.J., Saade G.R. (2020). Characteristics of Stillbirths Associated with Diabetes in a Diverse U.S. Cohort. Obstet. Gynecol..

[17] Dudley D.J. (2007). Diabetic-associated stillbirth: Incidence, pathophysiology, and prevention. Obstet. Gynecol. Clin. North Am..

[18] Vääräsmäki M., Pouta A., Elliot P., Tapanainen P., Sovio U., Ruokonen A., Hartikainen A.L., McCarthy M., Järvelin M.R. (2009). Adolescent manifestations of metabolic syndrome among children born to women with gestational diabetes in a general-population birth cohort. Am. J. Epidemiol..

[19] Bjornstad P., Chao L.C., Cree-Green M., Dart A.B., King M., Looker H.C., Magliano D.J., Nadeau K.J., Pinhas-Hamiel O., Shah A.S. (2023). Youth-onset type 2 diabetes mellitus: An urgent challenge. Nat. Rev. Nephrol..

[20] Vounzoulaki E., Khunti K., Abner S.C., Tan B.K., Davies M.J., Gillies C.L. (2020). Progression to type 2 diabetes in women with a known history of gestational diabetes: Systematic review and meta-analysis. BMJ.

[21] Varner M.W., Rice M.M., Landon M.B., Casey B.M., Reddy U.M., Wapner R.J., Rouse D.J., Tita A.T.N., Thorp J.M., Chien E.K. (2017). Pregnancies After the Diagnosis of Mild Gestational Diabetes Mellitus and Risk of Cardiometabolic Disorders. Obstet. Gynecol..

[22] Iordachescu A.C., Cirstoiu M.M., Zugravu C.A., Teodor O.M., Turcan N., Ducu I., Bohiltea R.E. (2020). Dietary behavior during pregnancy. Exp. Ther. Med..

[23] Kramer C.K., Campbell S., Retnakaran R. (2019). Gestational diabetes and the risk of cardiovascular disease in women: A systematic review and meta-analysis. Diabetologia.

[24] Wang Y.X., Mitsunami M., Manson J.E., Gaskins A.J., Rich-Edwards J.W., Wang L., Zhang C., Chavarro J.E. (2023). Association of Gestational Diabetes with Subsequent Long-Term Risk of Mortality. JAMA Intern. Med..

[25] Weisz B., Shrim A., Homko C.J., Schiff E., Epstein G.S., Sivan E. (2005). One hour versus two hours postprandial glucose measurement in gestational diabetes: A prospective study. J. Perinatol..

[26] Sandu C., Bica C., Salmen T., Stoica R., Bohiltea R., Gherghiceanu F., Pacu I., Stefan S., Serafinceanu C., Stoian A.P. (2021). Gestational diabetes—Modern management and therapeutic approach (Review). Exp. Ther. Med..

[27] Galindo R.J., Umpierrez G.E., Rushakoff R.J., Basu A., Lohnes S., Nichols J.H., Spanakis E.K., Espinoza J., Palermo N.E., Awadjie D.G. (2020). Continuous Glucose Monitors and Automated Insulin Dosing Systems in the Hospital Consensus Guideline. J. Diabetes Sci. Technol..

[28] Feig D.S., Donovan L.E., Corcoy R., Murphy K.E., Amiel S.A., Hunt K.F., Asztalos E., Barrett J.F.R., Sanchez J.J., de Leiva A. (2017). Continuous glucose monitoring in pregnant women with type 1 diabetes (CONCEPTT): A multicentre international randomised controlled trial. Lancet.

[29] Stirban A. (2013). Noninvasive Skin Fluorescence Spectroscopy for Diabetes Screening. J. Diabetes Sci. Technol..

[30] Boersma H.E., van der Klauw M.M., Smit A.J., Wolffenbuttel B.H.R. (2022). A non-invasive risk score including skin autofluorescence predicts diabetes risk in the general population. Sci. Rep..

[31] Tanin Ö.Ş., Kara M., Engin-Üstün Y., Göçmen A.Y., Yalvaç E.S. (2021). Comparison of glucose degradation product and receptor levels in diabetic and normal pregnancy. J. Turk. Ger. Gynecol. Assoc..

[32] Cosson E., Gary F., Nguyen M.T., Bianchi L., Sandre-Banon D., Biri L., Jaber Y., Cussac-Pillegand C., Banu I., Chiheb S. (2019). Gradual increase in advanced glycation end-products from no diabetes to early and regular gestational diabetes: A case-control study. Diabetes Metab..

[33] Foussard N., Cougnard-Grégoire A., Rajaobelina K., Delcourt C., Helmer C., Lamireau T., Gonzalez C., Grouthier V., Haissaguerre M., Blanco L. (2019). Skin Autofluorescence of Pregnant Women with Diabetes Predicts the Macrosomia of Their Children. Diabetes.

[34] de Ranitz-Greven W.L., Kaasenbrood L., Poucki W.K., Hamerling J., Bos D.C., Visser G.H., Biesma D.H., Beulens J.W., de Valk H.W. (2012). Advanced glycation end products, measured as skin autofluorescence, during normal pregnancy and pregnancy complicated by diabetes mellitus. Diabetes Technol. Ther..

[35] de Ranitz-Greven W.L., Bos D.C., Poucki W.K., Visser G.H., Beulens J.W., Biesma D.H., de Valk H.W. (2012). Advanced glycation end products, measured as skin autofluorescence, at diagnosis in gestational diabetes mellitus compared with normal pregnancy. Diabetes Technol. Ther..

[36] Maury E., Savel J., Grouthier V., Rajaobelina K., Corvo L., Lorrain S., Gonzalez C., Gin H., Barberger-Gateau P., Rigalleau V. (2015). Is skin autofluorescence a marker of metabolic memory in pregnant women with diabetes?. Diabet. Med..

[37] Chen J.H., Lin X., Bu C., Zhang X. (2018). Role of advanced glycation end products in mobility and considerations in possible dietary and nutritional intervention strategies. Nutr. Metab..

[38] Rajaraman B., Ramadas N., Krishnasamy S., Ravi V., Pathak A., Devasena C.S., Swaminathan K., Ganeshprasad A., Kuppuswamy A.A., Vedantham S. (2022). Correction to: Hyperglycemia cause vascular inflammation through advanced glycation end products/early growth response-1 axis in gestational diabetes mellitus. Mol. Cell. Biochem..

[39] Prasad A., Bekker P., Tsimikas S. (2012). Advanced Glycation End Products and Diabetic Cardiovascular Disease. Cardiol. Rev..

[40] Du T., Brandl B., Hauner H., Skurk T. (2023). Skin Autofluorescence Mirrors Surrogate Parameters of Vascular Aging: An Enable Study. Nutrients.

[41] van Waateringe R.P., Fokkens B.T., Slagter S.N., van der Klauw M.M., van Vliet-Ostaptchouk J.V., Graaff R., Paterson A.D., Smit A.J., Lutgers H.L., Wolffenbuttel B.H.R. (2019). Skin autofluorescence predicts incident type 2 diabetes, cardiovascular disease and mortality in the general population. Diabetologia.

[42] Reurean-Pintilei D., Pantea Stoian A., Salmen T., Stoica R.-A., Mititelu-Tartau L., Lazăr S., Timar B. (2024). Associations between Skin Autofluorescence Levels with Cardiovascular Risk and Diabetes Complications in Patients with Type 2 Diabetes. Biomedicines.

[43] Mao Y., Hu W., Xia B., Liu L., Han X., Liu Q. (2022). Association Between Gestational Diabetes Mellitus and the Risks of Type-Specific Cardiovascular Diseases. Front. Public Health.

[44] Chen A., Tan B., Du R., Chong Y.S., Zhang C., Koh A.S., Li L.J. (2024). Gestational diabetes mellitus and development of intergenerational overall and subtypes of cardiovascular diseases: A systematic review and meta-analysis. Cardiovasc. Diabetol..

[45] Jinno M., Takeuchi M., Watanabe A., Teruya K., Hirohama J., Eguchi N., Miyazaki A. (2011). Advanced glycation end-products accumulation compromises embryonic development and achievement of pregnancy by assisted reproductive technology. Hum. Reprod..

[46] Singh R., Barden A., Mori T., Beilin L. (2001). Advanced glycation end-products: A review. Diabetologia.

[47] Goto M., Yamagishi S.I., Matsui T., Koide K., Takita H., Tokunaka M., Sekizawa A. (2022). Predictive ability of serum advanced glycation end products at 11 to 13 weeks of gestation for early-onset preeclampsia. AJOG Glob. Rep..

[48] Shi Y., Qian J., Zhang Q., Hu Y., Sun D., Jiang L. (2020). Advanced glycation end products increased placental vascular permeability of human BeWo cells via RAGE/NF-kB signaling pathway. Eur. J. Obstet. Gynecol. Reprod. Biol..

[49] McElwain C.J., Tuboly E., McCarthy F.P., McCarthy C.M. (2020). Mechanisms of Endothelial Dysfunction in Pre-eclampsia and Gestational Diabetes Mellitus: Windows into Future Cardiometabolic Health?. Front. Endocrinol..

[50] Gurbuz O., Yorgancı A., Ozgu-Erdinc A.S., Tasci Y. (2022). First trimester screening of serum advanced glycation end products levels of pregnant women who have risk factors for gestational diabetes and their obstetric outcomes: A preliminary case-control study. J. Obstet. Gynaecol. J. Inst. Obstet. Gynaecol..

[51] Li H., Dong A., Lv X. (2019). Advanced glycation end products and adipocytokines and oxidative stress in placental tissues of pregnant women with gestational diabetes mellitus. Exp. Ther. Med..

[52] Saucedo R., Ortega-Camarillo C., Ferreira-Hermosillo A., Díaz-Velázquez M.F., Meixueiro-Calderón C., Valencia-Ortega J. (2023). Role of Oxidative Stress and Inflammation in Gestational Diabetes Mellitus. Antioxidants.

[53] Berceanu C., Cîrstoiu M., Mehedințu C., Brătilă P., Berceanu S., Vlădăreanu S., Bohîlțea R., Brătilă E. (2016). Hormone deficiency and its impact on the lower urinary tract. Proceedings of the 13th National Congress of Urogynecology.

[54] Sisay M., Edessa D., Ali T., Mekuria A.N., Gebrie A. (2020). The relationship between advanced glycation end products and gestational diabetes: A systematic review and meta-analysis. PLoS ONE.

[55] American Diabetes Association (2020). 2. Classification and Diagnosis of Diabetes: Standards of Medical Care in Diabetes-2020. Diabetes Care.

[56] Lowe W.L., Scholtens D.M., Kuang A., Linder B., Lawrence J.M., Lebenthal Y., McCance D., Hamilton J., Nodzenski M., Talbot O. (2019). HAPO Follow-up Study Cooperative Research Group Hyperglycemia and Adverse Pregnancy Outcome Follow-up Study (HAPO FUS): Maternal Gestational Diabetes Mellitus and Childhood Glucose Metabolism. Diabetes Care.

[57] Scholtens D.M., Kuang A., Lowe L.P., Hamilton J., Lawrence J.M., Lebenthal Y., Brickman W.J., Clayton P., Ma R.C., McCance D. (2019). HAPO Follow-up Study Cooperative Research Group, & HAPO Follow-Up Study Cooperative Research Group Hyperglycemia and Adverse Pregnancy Outcome Follow-up Study (HAPO FUS): Maternal Glycemia and Childhood Glucose Metabolism. Diabetes Care.

[58] Josefson J.L., Scholtens D.M., Kuang A., Catalano P.M., Lowe L.P., Dyer A.R., Petito L.C., Lowe W.L., Metzger B.E. (2021). HAPO Follow-up Study Cooperative Research Group Newborn Adiposity and Cord Blood C-Peptide as Mediators of the Maternal Metabolic Environment and Childhood Adiposity. Diabetes Care.

[59] Landon M.B., Rice M.M., Varner M.W., Casey B.M., Reddy U.M., Wapner R.J., Rouse D.J., Biggio J.R., Thorp J.M., Chien E.K. (2015). Eunice Kennedy Shriver National Institute of Child Health and Human Development Maternal-Fetal Medicine Units (MFMU) Network Mild gestational diabetes mellitus and long-term child health. Diabetes Care.

[60] Tam W.H., Ma R.C.W., Ozaki R., Li A.M., Chan M.H.M., Yuen L.Y., Lao T.T.H., Yang X., Ho C.S., Tutino G.E. (2017). In Utero Exposure to Maternal Hyperglycemia Increases Childhood Cardiometabolic Risk in Offspring. Diabetes Care.

[61] Saccone G., Khalifeh A., Al-Kouatly H.B., Sendek K., Berghella V. (2020). Screening for gestational diabetes mellitus: One step versus two step approach. A meta-analysis of randomized trials. J. Matern. -Fetal Neonatal Med..

[62] Bartakova V., Kollarova R., Kuricova K., Sebekova K., Belobradkova J., Kankova K. (2016). Serum carboxymethyl-lysine, a dominant advanced glycation end product, is increased in women with gestational diabetes mellitus. Biomed. Pap. Med. Fac. Univ. Palacky Olomouc. Czech. Repub..

[63] Júnior J.P.L., Brescansin C.P., Santos-Weiss I.C.R., Welter M., de Souza E.M., Rego F.G.d.M., Picheth G., Alberton D. (2017). Serum Fluorescent Advanced Glycation End (F-AGE) products in gestational diabetes patients. Arch Endocrinol. Metab..

[64] Ahmad M.S., Kimhofer T., Ahmad S., AlAma M.N., Mosli H.H., Hindawi S.I., Mook-Kanamori D.O., Šebeková K., Damanhouri Z.A., Holmes E. (2017). Ethnicity and skin autofluorescence-based risk-engines for cardiovascular disease and diabetes mellitus. PLoS ONE.

[65] Smit A.J., van de Zande S.C., Mulder D.J. (2022). Skin autofluorescence as tool for cardiovascular and diabetes risk prediction. Curr. Opin. Nephrol. Hypertens..

[66] Mooldijk S.S., Lu T., Waqas K., Chen J., Vernooij M.W., Ikram M.K., Zillikens M.C., Ikram M.A. (2024). Skin autofluorescence, reflecting accumulation of advanced glycation end products, and the risk of dementia in a population-based cohort. Sci. Rep..

[67] Cavero-Redondo I., Soriano-Cano A., Álvarez-Bueno C., Cunha P.G., Martínez-Hortelano J.A., Garrido-Miguel M., Berlanga-Macías C., Martínez-Vizcaíno V. (2018). Skin Autofluorescence-Indicated Advanced Glycation End Products as Predictors of Cardiovascular and All-Cause Mortality in High-Risk Subjects: A Systematic Review and Meta-analysis. J. Am. Heart Assoc..

[68] Stirban A., Heinemann L. (2014). Skin Autofluorescence—A Non-invasive Measurement for Assessing Cardiovascular Risk and Risk of Diabetes. Eur Endocrinol..

[69] McIntyre N.J. (2024). Trend and Monitoring of Skin Autofluorescence in Patients Receiving Hemodialysis. Kidney Int. Rep..

[70] Martínez-García I., Cavero-Redondo I., Pascual-Morena C., Otero-Luis I., Fenoll-Morate M., Lever-Megina C.G., Rodríguez-Gutiérrez E., Saz-Lara A. (2025). Reference Values of Skin Autofluorescence by Age Groups in Healthy Spanish Adults: Results from the EVasCu Study, a Systematic Review, and a Meta-Analysis. J. Clin. Med..

[71] Sánchez E., Sánchez M., López-Cano C., Bermúdez-López M., Valdivielso J.M., Farràs-Sallés C., Pamplona R., Torres G., Mauricio D., Castro E. (2022). Is There a Link between Obesity Indices and Skin Autofluorescence? A Response from the ILERVAS Project. Nutrients.

[72] Reurean-Pintilei D., Pantea Stoian A., Potcovaru C.-G., Salmen T., Cinteză D., Stoica R.-A., Lazăr S., Timar B. (2024). Skin Autofluorescence as a Potential Adjunctive Marker for Cardiovascular Risk Assessment in Type 2 Diabetes: A Systematic Review. Int. J. Mol. Sci..

[73] Chen J., Waqas K., Tan R.C., Voortman T., Ikram M.A., Nijsten T.E.C., de Groot L.C., Uitterlinden A.G., Zillikens M.C. (2020). The association between dietary and skin advanced glycation end products: The Rotterdam Study. Am. J. Clin. Nutr..

[74] Atzeni I.M., Boersema J., Pas H.H., Diercks G.F.H., Scheijen J.L.J.M., Schalkwijk C.G., Mulder D.J., van der Zee P., Smit A.J. (2020). Is skin autofluorescence (SAF) representative of dermal advanced glycation endproducts (AGEs) in dark skin? A pilot study. Heliyon.

[75] Noordzij M.J., Lefrandt J.D., Graaff R., Smit A.J. (2011). Dermal Factors Influencing Measurement of Skin Autofluorescence. Diabetes Technol. Ther..

[76] Lutgers H.L., Graaff R., Links T.P., Ubink-Veltmaat L.J., Bilo H.J., Gans R.O., Smit A.J. (2006). Skin autofluoresce as a noninvasive marker of vascular damage in patients with type 2 diabetes. Diabetes Care.

[77] Simon Klenovics K., Kollárová R., Hodosy J., Celec P., Sebeková K. (2014). Reference values of skin autofluorescence as an estimation of tissue accumulation of advanced glycation end products in a general Slovak population. Diabet. Med. A J. Br. Diabet. Assoc..

[78] Alkhami F., Borderie G., Foussard N., Larroumet A., Blanco L., Barbet-Massin M.A., Ferriere A., Ducos C., Mohammedi K., Fawaz S. (2024). The skin autofluorescence may help to select patients with Type 2 diabetes candidates for screening to revascularization procedures. Cardiovasc. Diabetol..

[79] Chen J., Arshi B., Waqas K., Lu T., Bos D., Ikram M.A., Uitterlinden A.G., Kavousi M., Zillikens M.C. (2023). Advanced glycation end products measured by skin autofluorescence and subclinical cardiovascular disease: The Rotterdam Study. Cardiovasc. Diabetol..

[80] Boersma H.E., van Waateringe R.P., van der Klauw M.M., Graaff R., Paterson A.D., Smit A.J., Wolffenbuttel B.H.R. (2021). Skin autofluorescence predicts new cardiovascular disease and mortality in people with type 2 diabetes. BMC Endocr. Disord..

[81] Hebert J.F., Myatt L. (2021). Placental mitochondrial dysfunction with metabolic diseases: Therapeutic approaches. Biochim. Biophys. Acta-Mol. Basis Dis..

[82] Di Fabrizio C., Giorgione V., Khalil A., Murdoch C.E. (2022). Antioxidants in Pregnancy: Do We Really Need More Trials?. Antioxidants.

[83] Hagen J.M., Cornelissen A., Veeneman R.R., van der Heijden H.S., Sutterland A.L., Vermeulen J.M., Tan H.L., de Haan L. (2025). Skin advanced glycation end products, indicating cumulative oxidative stress, associated with schizophrenia but not with psychosis-like experiences. Schizophr. Res..

[84] Chen W., Zhang Y., Yue C., Ye Y., Chen P., Peng W., Wang Y. (2017). Accumulation of Advanced Glycation End Products Involved in Inflammation and Contributing to Severe Preeclampsia, in Maternal Blood, Umbilical Blood and Placental Tissues. Gynecol. Obstet. Investig..

[85] Wierzchowska-Opoka M., Grunwald A., Rekowska A.K., Łomża A., Mekler J., Santiago M., Kabała Z., Kimber-Trojnar Ż., Leszczyńska-Gorzelak B. (2023). Impact of Obesity and Diabetes in Pregnant Women on Their Immunity and Vaccination. Vaccines.

[86] Nelson B.N., Friedman J.E. (2024). Developmental Programming of the Fetal Immune System by Maternal Western-Style Diet: Mechanisms and Implications for Disease Pathways in the Offspring. Int. J. Mol. Sci..

[87] Eleftheriades A., Koulouraki S., Belegrinos A., Eleftheriades M., Pervanidou P. (2025). Maternal Obesity and Neurodevelopment of the Offspring. Nutrients.

[88] Marschallinger J., Iram T., Zardeneta M., Lee S.E., Lehallier B., Haney M.S., Pluvinage J.V., Mathur V., Hahn O., Morgens D.W. (2020). Lipid-droplet-accumulating microglia represent a dysfunctional and proinflammatory state in the aging brain. Nat. Neurosci..

[89] Jankowska M., Szadkowska A., Pietrzak I., Chrzanowski J., Sołek J., Fendler W., Mianowska B. (2024). Assessment of Skin Autofluorescence and Its Association with Glycated Hemoglobin, Cardiovascular Risk Markers, and Concomitant Chronic Diseases in Children with Type 1 Diabetes. Nutrients.

[90] Zavorins A., Silova A., Voicehovska J., Kisis J. (2019). Rubeosis faciei diabeticorum is not associated with oxidative stress and skin autofluorescence. An. Bras. Dermatol..

[91] Mustață M.-L., Neagoe C.-D., Rădulescu V.-M., Dragne I.-G., Cîmpeanu R.-C., Radu L., Ahrițculesei R.-V., Forțofoiu D., Predoi M.-C., Ianoși S.-L. (2025). Association Between Systemic Inflammation, Metabolic Syndrome and Quality of Life in Psoriasis Patients. Life.

[92] Metcalfe A., Sabr Y., Hutcheon J.A., Donovan L., Lyons J., Burrows J., Joseph K.S. (2017). Trends in Obstetric Intervention and Pregnancy Outcomes of Canadian Women with Diabetes in Pregnancy from 2004 to 2015. J. Endocr. Soc..

[93] Rajaobelina K., Farges B., Nov S., Maury E., Cephise-Velayoudom F.L., Gin H., Helmer C., Rigalleau V. (2017). Skin autofluorescence and peripheral neuropathy four years later in type 1 diabetes. Diabetes/Metab. Res. Rev..

[94] Mustață M.-L., Ionescu M., Radu L., Neagoe C.-D., Ahrițculesei R.-V., Cîmpeanu R.-C., Matei D., Amzolini A.-M., Predoi M.-C., Ianoși S.-L. (2024). The Role of Metabolic Syndrome in Psoriasis Treatment Response: A One-Year Comparative Analysis of PASI Progression. Diagnostics.

[95] Edmonds L.K., Kandasamy Y., Lamont A., O’Connor S. (2012). Perinatal arterial ischemic stroke in northern Queensland. Am. J. Perinatol..

[96] Said A.S., Manji K.P. (2016). Risk factors and outcomes of fetal macrosomia in a tertiary centre in Tanzania: A case-control study. BMC Pregnancy Childbirth.

[97] Nielsen L.R., Ekbom P., Damm P., Glümer C., Frandsen M.M., Jensen D.M., Mathiesen E.R. (2004). HbA1c levels are significantly lower in early and late pregnancy. Diabetes Care.

